# Connecting the Dots Between Mindset and Impostor Phenomenon, via Fear of Failure and Goal Orientation, in Working Adults

**DOI:** 10.3389/fpsyg.2021.588438

**Published:** 2021-11-16

**Authors:** Rebecca Noskeau, Angeli Santos, Weiwei Wang

**Affiliations:** Division of Psychiatry and Applied Psychology, School of Medicine, University of Nottingham, Nottingham, United Kingdom

**Keywords:** mindset, implicit theories, impostor phenomenon, impostor syndrome, fear of failure, goal orientation, goal setting, serial-parallel mediation

## Abstract

This study aims to investigate the relationship between mindset and impostor phenomenon, via the explanatory role of fear of failure and goal orientation in the work domain. Only one known study has previously connected mindset and impostor phenomenon in the scientific literature among females in a university setting. Data was collected from 201 working adults, with a roughly equal male-female ratio, from a range of sectors in the United Kingdom, Ireland, and United States. Participants completed an online survey comprising the Implicit Theories of Intelligence Scale, the Performance Failure Appraisal Inventory, Work Domain Goal Orientation Instrument, and the Clance Impostor Phenomenon Scale (CIPS). We tested a serial-parallel mediation model using structural equation modeling. The results suggest that people with a fixed mindset tend to experience more impostor phenomenon at work and this relationship is predominantly explained by their fear of failure. Further, when employees are also motivated by a performance avoid goal orientation, the relationship increases in strength. This indirect relationship suggests that staff training, and coaching interventions designed to increase people’s belief that they can develop their abilities results in a reduction of their fear of failure and in their motivation to want to avoid showing their inability at work. The results also suggest cultivating environments that promote a growth mindset and learning goal orientation, alongside the safety to fail, could lessen the negative effects of having a fixed mindset, reduce fear of failure, and alleviate impostor phenomenon’s negative impact on employee career development and wellbeing.

## Introduction

### Mindset

Mindset refers to the beliefs individuals have about their abilities and whether they perceive them as being more innate and “fixed,” or more malleable and capable of “growth” (Dweck, 2000). Dweck (2017) suggests everyone possesses a mixture of a fixed and growth mindset, varying across different contexts such as work and home; and attributes such as personality and intelligence. Research with children found that having a predominantly fixed mindset predicted negative affect concurrently and at 7 months (King, 2017). Further, a meta-analysis revealed a relationship between fixed mindsets and more pronounced mental health problems in youths (Schleider et al., 2015). Mindset theory proposes that fixed mindsets can be evoked through situational factors, such as perceived threat and challenge (Dweck, 2017). Even well-meaning praise, for example, such as complimenting someone on being smart or talented, is capable of reinforcing fixed beliefs and behaviors (Mueller and Dweck, 1998). Blackwell et al. (2007) reported that fixed mindsets were associated with poorer achievement outcomes; whilst growth mindsets predicted increased motivation, resilience, and effort in the face of setbacks – leading to better performance overall.

Despite the popularity and prevalence of mindset theory in recent years, the validity of mindset research and the effects of mindset on performance have been heavily called into question (Denworth, 2019). Researchers have found that mindset does not predict or improve academic performance in the form of scholastic aptitude (Bahník and Vranka, 2017). Further, Li and Bates (2019) could not reproduce the relationships between mindset and academic performance, praise, response to failure, or progress in school, as reported by Blackwell et al. (2007). In educational settings, many of the studies exploring mindset have demonstrated that mindset interventions matter most when children are at risk, yet have little to no impact on performance for the general student population (Yeager et al., 2016, 2019; Sisk et al., 2018).

A randomized controlled trial by Yeager et al. (2019) highlighted the influential moderating role of the social environment on the efficacy of growth mindset interventions. They found the effects of their intervention on academic performance were more sustained when students’ peer norms aligned with messages of the intervention and supported students taking on academic challenges. This research revealed a broader beneficial impact of growth mindset interventions beyond grades. All children who received the intervention were more likely to choose the advanced math course the following year. The latter suggests that mindset training supports development by increasing one’s motivation to pursue challenges (Yeager et al., 2019). In line with the broader beneficial view of mindset interventions, a randomized trial found that a 45-min computerized growth mindset intervention significantly reduced depressive symptoms in rural adolescent females at baseline and 4 months later (Schleider et al., 2020). The questions now posed in mindset research are, does mindset matter for performance and wellbeing outcomes? If so, for whom, how and when is this the case?

### Impostor Phenomenon

Impostor phenomenon is defined as a “pervasive psychological experience of a person believing they are a self-perceived intellectual fraud and fearing they may be recognized as an impostor” (Sakulku and Alexander, 2011). In the academic literature, the term “impostor phenomenon” is used, however, in the lay literature, the term “imposter syndrome” is more common (Bravata et al., 2020). A systematic review demonstrated that impostor phenomenon is prevalent among women, men, and multiple ethnic groups (Bravata et al., 2020).

Despite impostors often achieving impressive successes (Clance and Imes, 1978), they live in perpetual fear of being unmasked as a fraud (Matthews and Clance, 1985). Clance and O’Toole (1987, p. 4) show that when a performance challenge is presented it triggers the impostor cycle. Fear, doubt, and anxiety are experienced, and questions are asked as to whether success is possible “this time around.” This is met with either over-preparation and perfectionism, or procrastination followed by frenzied preparation (Clance and O’Toole, 1987). When successful outcomes are achieved, over-preparation confirms the belief that such an outcome was only attained through all-consuming effort, thus, reinforcing the need to perfectly prepare the next time. Conversely, procrastination followed by frenzied preparation feeds into an impostor’s tendency to attribute success to luck or effort. Both approaches prevent impostors from attributing successes to ability and reinforce self-doubt (Clance and O’Toole, 1987).

Irrespective of performance outcomes, impostors are more likely to make attributions to inability (Cozzarelli and Major, 1990). However, this pattern of external attribution does not carry over to social situations, underscoring the importance of context for impostor thought processes (Brauer and Wolf, 2016). Impostors are concerned with how their ability compares with others (Kumar and Jagacinski, 2006). They view others as being more capable and smarter than they are, overestimate others’ abilities, and underestimate how much effort others invest in their successes; whilst simultaneously attributing their own need to exert effort to achieve success as being indicative of inability (Harvey, 1982; Clance and O’Toole, 1987; Kumar and Jagacinski, 2006; Parkman, 2016). It therefore follows, that impostors are prone to discounting their successes by attributing them to non-ability factors such as luck, fooling others and effort (Clance and Imes, 1978). Contrary to this literature connecting attributions of increased personal effort to higher impostor feelings, Vaughn et al. (2020) found that attributing success to effort was related to decreased impostor feelings in female academics. Thus, highlighting *how* effort is conceptualized and attributed by impostors is critical.

Impostors’ perfect-procrastinate approaches and attributional styles result in an inability to internalize or grow from their achievements (Harvey and Katz, 1985). Instead, they appear stuck in a pattern of continually underestimating themselves and undervaluing their abilities (Clance and O’Toole, 1987). As a consequence, impostors are more likely to suffer from low self-esteem and other psychological issues, such as depression and anxiety (Sakulku and Alexander, 2011; Bravata et al., 2020). Consequently, Bravata et al. (2020) call for the impostor syndrome to be classified as a disorder and propose that evidence-based education and therapeutic interventions are needed to alleviate this psychological impact and better support those struggling with impostor feelings. However, a recent study has discovered having impostor thoughts is not all bad. Tewfik (2021) identified impostor thoughts can be a motivator that is good for job mastery and can improve interpersonal performance at work. These findings lead to questions as to whether impostor feelings are indeed harmful or helpful to growth and what can be done to alleviate the harmful psychological impacts demonstrated by prior research.

### Fixed Mindsets and Impostors

Both fixed mindsets and impostors seemingly operate from a pervasive fixed sense of self and ability, impacting wellbeing and motivation (Sakulku and Alexander, 2011; Dweck, 2017). Impostors demonstrate characteristics suggesting they have fixed mindsets (Kumar and Jagacinski, 2006). An impostor’s core fear that they will be unmasked as an intellectual fraud (Matthews and Clance, 1985) highlights their preoccupation with others finding out they are less capable than presently perceived. Kumar and Jagacinski (2006) propose this preoccupation may originate from the belief their abilities are fixed. For, if they believed they were capable of development, their abilities could be improved through effort, thus diminishing their core fears. Further, fixed mindsets and impostors share several commonalities in performance and achievement settings. Fixed mindsets and impostors tend to see the need for effort as signaling a lack of inherent ability (Clance and Imes, 1978; Blackwell et al., 2007), and generalize a single instance of failure to their being a failure (Thompson et al., 1998; Dweck, 2017).

In the lay literature, a Google Search for “fixed mindset and impostor” returns 150,000 results. However, to date, only one known peer-reviewed study has demonstrated a connection between fixed mindsets and impostors within a small sample (*n* = 42) of female university students (Kumar and Jagacinski, 2006). Kumar and Jagacinski (2006) found that women who believed intelligence was a “fixed” entity were more likely to report having impostor fears. This study builds upon this research and connects the dots between mindset and impostor phenomenon in the work domain, via the following interrelationships discussed below.

### Mindset and Fear of Failure

Fear of failure is defined as the “tendency to appraise threat and feel anxious during situations that involve the possibility of failing” (Conroy et al., 2007). Individuals with fixed mindsets are more prone to internalizing failures and overgeneralizing them to their global self-concepts, such that, instead of thinking “I failed,” they think “I am a failure” (Dweck, 2017). Researchers have shown how one’s beliefs about their abilities (mindset), predicts subsequent efforts following failure (Hong et al., 1999). Blackwell et al. (2007) reported that individuals with fixed mindsets perceive the need for effort as indicative of their inability, which leads them to adopt maladaptive strategies, like withdrawing effort and avoiding challenges, to protect themselves from failing. This approach, paradoxically, results in failure, similar to a self-fulfilling prophecy. Conversely, those with growth mindsets employ effort as a mechanism for developing ability and mastery, resulting in their achievement of success through adaptive and remedial actions in response to failures (Hong et al., 1999).

Seminal mindset and goal orientation research have identified two over-arching response patterns when individuals face challenges (Dweck and Leggett, 1988). One pattern, identified as a “helpless response” which later evolved into an aspect of fixed mindset, is characterized by an avoidance of challenges and a deterioration in performance when faced with obstacles (Dweck and Leggett, 1988; Dweck, 2017). In contrast, the other pattern, known as a “mastery response,” which later evolved into an aspect of a growth mindset, involves seeking challenges alongside employing effective strategies and resilience in the face of failures (Dweck and Leggett, 1988; Dweck, 2017). What is interesting here, is that the seminal researchers revealed that mastery-orientated individuals did not appraise setbacks as “failures,” but instead, viewed them as part and parcel of the learning process (Dweck and Leggett, 1988). They were able to keep forging forward by focusing their attention on effective learning strategies, thus enabling them to develop their abilities. In stark contrast, individuals with a helpless response were so consumed by their self-perceived failures, and their beliefs that these failures underscored the limits of their fixed abilities, that it prevented them from activating crucial learning strategies. This, in turn, stopped them from creating opportunities and accessing resources to develop their abilities (Dweck and Leggett, 1988). Therefore, it could be said that whilst those with fixed mindsets fixate on failures; those with growth mindsets are able to grow through and beyond them.

### Fear of Failure and Impostor Phenomenon

Impostors are believed to be driven by a core need to avoid failure at all costs (Clance and O’Toole, 1987). This is supported by quantitative research that demonstrated that fear of failure predicted impostor phenomenon in a sample of university students (Neureiter and Traut-Mattausch, 2017); and qualitative research which explored how impostor feelings, driven by fear of failure, stifles career advancement in female academics (Howe-Walsh and Turnbull, 2016). Impostors attribute failure to stable internal factors and overgeneralize one instance of failure to their global self-concept (Thompson et al., 1998). Consequently, impostors are believed to live in a continual “*dread of evaluation”* and “*terror of failure”* (Clance and O’Toole, 1987, p. 4; Cozzarelli and Major, 1990, p. 416).

Impostor thoughts are related to fear of social exposure and attracting negative judgments from others (Thompson et al., 2000). Research has related impostor phenomenon to social anxiety via an impostor’s fear of negative evaluation, need for social recognition, and preoccupation with what others think about them (Chrisman et al., 1995). Thompson et al. (2000) found that impostors have higher perfectionistic concerns over making mistakes; perceive they make more mistakes; feel worse about making mistakes; and, have less positive mood before and after performance situations. Within an examination context, it was found that impostors believed they would perform less well, experienced more anxiety, exhibited more negative responses to subjective failure, and expressed greater dissatisfaction with their performance, as compared with non-impostors (Cozzarelli and Major, 1990). Consequently, impostors are unable to defend themselves psychologically against the negative consequences of failing (Cozzarelli and Major, 1990). Nevertheless, impostors’ fears about failing are unfounded in terms of performance differences between impostors and non-impostors – meaning that this is a difference in perception of ability and not capability itself (Cozzarelli and Major, 1990; Thompson et al., 2000).

### Mindset and Goal Orientation

“Goal orientations define why and how people are trying to achieve various objectives and refer to overarching purposes of achievement behavior” (Kaplan and Maehr, 2007, p. 142). They are mental frameworks individuals use to filter and understand information, create meaning and purpose, construct and appraise situations, and take actions from, within achievement settings (Brett and Vandewalle, 1999; Kaplan and Maehr, 2007). In their review, Vandewalle et al. (2019) highlighted evidence for dispositional (an individual’s characteristic style) and state (the characteristics of the situation) forms of goal orientation, which, when aligned, can enhance task performance (Seijts et al., 2004). Predominant research in goal orientation broadly identifies two overarching types: performance (ability/ego) and learning (mastery/task); broken down into three subtypes (Vandewalle et al., 2019). The Work Domain Goal Orientation Instrument utilized for this study (Vandewalle, 1997, p. 1000; Brett and Vandewalle, 1999) operationalizes and assesses three subtypes of goal orientations:

1. Performance prove goal orientation (prove GO): Motivated by desires to prove competence and gain favorable judgments about it.
2. Performance avoid goal orientation (avoid GO): Motivated by desires to avoid disproving competence and avoid negative judgments about it.
3. Learning goal orientation (learning GO): Motivated by the “self-referenced” (Nicholls, 1984, p. 329) desires to improve their competence, and develop themselves through the attainment of new skills and the mastery of new situations.

Researchers “quite unequivocally” suggest that a learning goal orientation is an adaptive motivational orientation producing many benefits (Kaplan and Maehr, 2007, p. 170); including better engagement, deeper-level processing and learning, seeking challenges and feedback, persistence, increased productivity, metacognition, and an overall more adaptive orientation toward life, exhibited through increased wellbeing and more positive feelings about oneself and others (Kaplan and Maehr, 2007; Vandewalle et al., 2019). A meta-analysis revealed that performance and learning goal orientations have largely the same impact on academic performance (Linnenbrink-Garcia et al., 2008). However, citing decades of research, Vandewalle et al. (2019) showed how performance goal orientations can become problematic for performance-related activities and outcomes (such as feedback-seeking, effort use, goal content and task performance) when fear of failure and complexity are high; and when self-efficacy and ability are low. Further, they showed that a learning goal orientation can become advantageous, and even protective (as in the cases of low self-efficacy and negative feedback) for performance-related activities and outcomes; the exception being when task complexity and/or rule consistency are low. In this instance, Vandewalle et al. (2019), suggest that individuals with performance goal orientations outperform learning goal orientations.

Researchers have linked performance goal orientations with fixed mindsets and learning goal orientations with growth mindsets (Dweck and Leggett, 1988; Cury et al., 2006). Those with fixed mindsets and performance goal orientations appear to share beliefs about ability and effort. Namely, that developing abilities is difficult, that ability is the most important determinant for achievement, that high ability individuals do not have to use as much effort to succeed, and finally, that the need to exert effort can signal inability (Dweck and Leggett, 1988; Vandewalle et al., 2019). The correlates found between the two have led researchers to suggest that a fixed mindset leads to a performance goal orientation (Dweck and Leggett, 1988). Conversely, those with learning goal orientations and growth mindsets appear to share beliefs that effort is a more important determinant of achievement than ability; that abilities can be developed over time through effort, and that effort is a tool to develop ability, and ultimately performance (Dweck and Leggett, 1988; Vandewalle et al., 2019). These correlates led researchers to suggest that a growth mindset results in a learning goal orientation (Dweck and Leggett, 1988).

The theoretical models utilized in this study are based on research linking performance-related goal orientations with fixed mindsets and learning-related goal orientations with growth mindsets. However, Vandewalle et al. (2019) highlighted concerns regarding inconsistent empirical evidence regarding these causal correlates, as exemplified by Kumar and Jagacinski (2006) findings that mindset only related to goal orientation in female undergraduates. This study adds to the body of empirical evidence exploring these correlates.

### Fear of Failure and Goal Orientation

Researchers have demonstrated key patterns between fear of failure and goal orientation. Using their achievement goal model, Elliot and Church (1997) demonstrated that performance-avoidance goal orientation is related to fear of failure, and that mastery-achievement goal orientation is related to the need for achievement, whilst the performance-approach goal orientation is related to both. In the work domain, fear of negative evaluation was found to be positively related to prove and avoid GOs, and negatively related to learning GOs (Vandewalle, 1997). Indeed, fear of failure predicted both performance-approach and performance-avoid goals among undergraduate students (Elliot and McGregor, 1999). Further, within a student sports setting, fear of failure predicted the use of performance-avoidance GO; both preceding and increasing the probability of their use (Conroy and Elliot, 2004). However, contrary to prior research and expectations, Conroy and Elliot (2004) found that fear of failure was not an antecedent or outcome of performance-approach goals, despite their being positively related. Finally, a null relationship was reported between mastery goals and fear of failure (Elliot and McGregor, 1999).

Vandewalle’s (1997) performance prove, performance avoid and learning goal orientation subscales have respective theoretical parallels with Elliot and Church’s (1997) performance-approach, performance-avoid and mastery achievement goal orientation subscales. However, the two are conceptualized and operationalized differently. Vandewalle’s instrument conceptualizes goal orientation as dispositional and situated at a distal motivational level; whereas Elliot and Church define goal orientation as a dispositional outcome related to the need for achievement and fear of failure; the antecedent of which is perceived ability (Vandewalle et al., 2019). Nevertheless, the overarching theoretical parallels between these two instruments provide tentative theoretical support for the predicted interrelationships investigated in this study.

### Goal Orientation and Impostor Phenomenon

Research has also demonstrated several parallels between goal orientation and impostor feelings. Individuals with performance (prove/avoid) goal orientations and impostors are prone to viewing effort as a potential signal of inability (Nicholls, 1984; Clance and O’Toole, 1987; Kumar and Jagacinski, 2006). Further, they both tend to attribute a single failure to their global self-concepts (Kumar and Jagacinski, 2006). Moreover, they are both concerned with how their ability compares with others and how they are viewed by them; exhibited through their fear of negative evaluation, need for social recognition and preoccupation with what others think about them (Chrisman et al., 1995; Kumar and Jagacinski, 2006; Vandewalle et al., 2019).

Prove GOs can be related to an impostor’s need for perfectionism and superior performance (Sakulku and Alexander, 2011); thus, potentially manifesting in the over-preparation phase of the impostor cycle (Clance and O’Toole, 1987). Avoid GOs align with an impostor’s need to avoid failure at all costs which could manifest in the procrastinate then frenzied preparation phase of the impostor cycle (Clance and O’Toole, 1987). In contrast, learning GOs appear to be theoretically contrary to an impostor’s motivational attributes, therefore, a learning GO is theorized as having a negative relationship with impostor phenomenon. Using the Patterns of Adaptive Learning Scales (Midgley et al., 1998) Kumar and Jagacinski (2006) demonstrated these relationships with notable gender differences in university students. Namely, that impostor fears were positively related to performance ability-approach and ability-avoid goals for men; whilst for women, impostor fears were positively related to ability-approach goals and negatively related to task goals.

It is worth noting, however, that the Work Domain Goal Orientation Instrument (Vandewalle, 1997) utilized in this study is conceptualized and operationalized differently from the Patterns of Adaptive Learning Scales (Midgley et al., 1998). Vandewalle’s performance prove, performance avoid and learning subscales, can, however, be broadly theoretically overlain upon Midgley et al.’s (1998) ability-approach, ability-avoid and task goal subscales, respectively. Although broad theoretical parallels are being drawn, the two models of goal orientation are different at the construct level. The Work Domain Goal Orientation Instrument conceptualizes goal orientation as dispositional and at the distal motivational level of workers (Vandewalle et al., 2019), whereas the Patterns of Adaptive Learning Scales defines goal orientation in terms of achievement goals being motivators of achievement behavior in students (Midgley et al., 1998). Nonetheless, due to the overarching conceptual parallels, it is proposed that Kumar and Jagacinski’s (2006) findings lend tentative theoretical support for the interrelationships explored here.

### Significance of Connecting Mindset to Impostor Phenomenon via Fear of Failure and Goal Orientation in the Work Domain

Fixed mindsets and impostor feelings impact wellbeing and motivation (Sakulku and Alexander, 2011; Dweck, 2017). The characteristics evident in impostors and individuals with a fixed mindset are also evident in those with performance goal orientations (Nicholls, 1984; Clance and O’Toole, 1987; Kumar and Jagacinski, 2006).

Vandewalle et al. (2019) highlighted that a strong learning goal orientation is beneficial for several distal outcomes important to the work domain, including: performance; leadership development and style; wellbeing; openness; adjustment to change; and organizational citizenship behaviors. Furthermore, researchers have demonstrated that it is possible to train people to utilize a learning goal orientation and learning goals, amongst students (Stevens and Gist, 1997; Kozlowski et al., 2001) and unemployed job seekers (Van Hooft and Noordzij, 2009; Noordzij et al., 2013), leading to more beneficial behaviors and outcomes for both. Vandewalle et al. (2019) outlined strategies based on prior research which leaders can use to promote and strengthen a learning goal orientation amongst their employees. Relatedly, Seijts et al. (2004) demonstrated that effective goal setting is a situational variable that can mask differences in performance, originating from a dispositional goal orientation. They demonstrated that a specific high learning goal, as opposed to a high-performance goal, can produce higher performance on a complex task requiring the acquisition of ability and skill – an effect on performance which is enhanced when individuals possess a dispositional learning goal orientation (Seijts et al., 2004). Lastly, a relationship between experiencing fewer impostor feelings and having the ability to set very clear goals was identified by Sanford et al. (2015).

Taking these findings collectively, the following is proposed. If fixed mindsets are related to dispositional performance goal orientations and growth mindsets are related to dispositional learning goal orientations, then organizations that effectively utilize goal setting and consciously cultivate learning goal orientations and growth mindsets, may be able to override differences originating from performance goal orientations, and ultimately, fixed mindsets. Therefore, cultivating growth mindsets and learning goal orientations through effective goal setting, may have the potential to disrupt the connections between fixed mindsets and impostor feelings, operating via fear of failure and goal orientation.

#### Aims and Hypotheses

**FIGURE 1:**
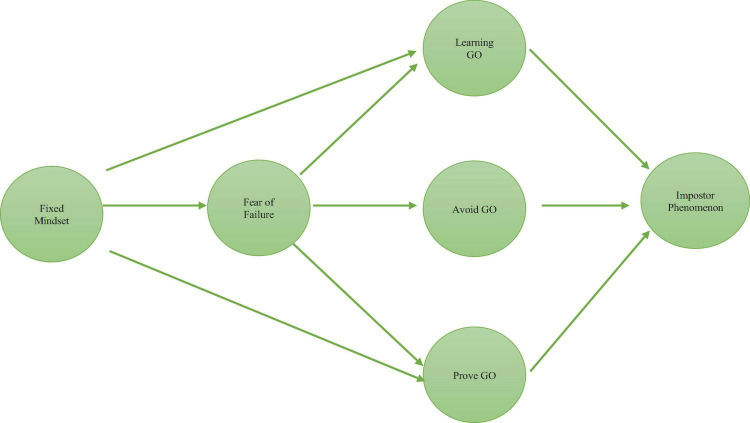
The conceptual model.

This study has two main aims. Firstly, to investigate the simple relationships^1^ between mindset, fear of failure, goal orientations and impostor phenomenon, covered by hypotheses 1–6. Secondly, this study seeks to deconstruct the indirect relationship between mindset and impostor phenomenon, by way of fear of failure and goal orientation, using a serial-parallel mediation model depicted in Figure 1. We then hypothesize two indirect mechanisms. Hypothesis 7 treats fear of failure and goal orientation as parallel mediators which may covary. Hypothesis 8 assumes a causal relationship leading from fear of failure to goal orientation, which therefore yields three possible paths. The full set of hypotheses for the study are as follows:

#### Simple Relationships

Hypothesis 1 (H1): Fixed mindset will be positively related to impostor phenomenon.

Hypothesis 2 (H2): Fixed mindset will be positively related to fear of failure.

Hypothesis 3 (H3): Fixed mindset will be negatively related to learning GO (H3a), positively related to prove GO (H3b) and positively related to avoid GO (H3c).

Hypothesis 4 (H4): Fear of failure will be positively associated with impostor phenomenon.

Hypothesis 5 (H5): GOs will be related to impostor phenomenon, in that, a learning GO will be negatively related to impostor phenomenon (H5a); prove GO will be positively related to impostor phenomenon (H5b); and avoid GO will be positively related to impostor phenomenon (H5c).

Hypothesis 6 (H6): Fear of failure will be negatively related to Learning GO (H6a); positively related to prove GO (H6b); and positively related to avoid GO (H6c).

#### Indirect Effects

Hypothesis 7 (H7): The relationship between mindset and impostor phenomenon is mediated in parallel by fear of failure (H7a), learning GO (H7b), prove GO (H7c) and avoid GO (H7d).

Hypothesis 8 (H8): The relationship between mindset and impostor phenomenon is mediated in a serial-parallel fashion with three paths: fear of failure and learning GO in serial (H8a); fear of failure and prove GO in serial (H8b); and fear of failure and avoid GO in serial (H8c).

## Materials and Methods

### Participants and Procedure

We used a self-report, cross-sectional, between-subjects design. Ethical approval was granted by the Ethics Committee of the Division of Psychiatry and Applied Psychology, University of Nottingham, United Kingdom. The electronic survey was distributed to potential participants using JISC (2019) with the help of gatekeepers from 22 participating organizations in the United Kingdom, United States, and Ireland. After 12 weeks, 211 responses were collected from the following sectors: Gaming, Finance, Travel, Education, Industrial, Automobile, Utilities, Retail, IT and Tech, Customer Service, and Recruitment. 10 cases were removed due to data cleaning procedures (two cases had data points missing not at random and eight cases were identified as multivariate outliers). Consequently, we had 201 valid samples. Ninety four respondents identified as female (46.8%), 94 identified as male (46.8%) and 13 (6.5%) respondents did not provide their gender. Ages ranged from 21 to 67 years (*M* = 40.43, *SD* = 12.11). The average number of “years at current organization” (*M* = 7.87, *SD* = 8.19, *Md* = 5.00) and “years in current role” (*M* = 4.51, *SD* = 6.08, *Md* = 2.00) ranged from 0 to 42 years.

### Instruments

#### Mindset

Mindset was measured using the Implicit Theories of Intelligence Scale: Self-Form for Adults (Dweck, 2000). Participants rate their beliefs on the fixedness and malleability of their intelligence using a 6-point Likert scale, ranging from “strongly disagree” to “strongly agree.” This eight-item scale contains four items for fixed (entity) mindset and four for growth (incremental) mindset, such as: “*You have a certain amount of intelligence, and you can’t really do much to change it”* and “*No matter who you are, you can significantly change your intelligence leve*l”. The literature has documented good overall internal consistency (α = 0.82–0.97) and evidence supporting its construct validity (Dweck et al., 1995). Our confirmatory factor analysis (CFA) showed a good fit for a unidimensional structure of this scale with an alpha value of 0.95 in the current sample.

#### Fear of Failure

Fear of failure was assessed using the 25-item Long-Form Performance Failure Appraisal Inventory (Conroy et al., 2002), which has good validity and reliability exceeding α = 0.80 (Conroy et al., 2003). The fear of failure scale is composed of five subscales: fear of experiencing shame and embarrassment (seven-items), fear of devaluing one’s self-estimate (four-items), fear of having an uncertain future (four-items), fear of important others losing interest (five-items) and fear of upsetting important others (five-items). Participants indicated the frequency with which they believed each statement was true for them using a 5-point Likert scale ranging from −2 (do not believe at all) to +2 (believe 100% of the time), with a midpoint of 0 (believe 50% of the time). Example statements are: *“When I am failing, it is often because I am not smart enough to perform successfully”* and *“When I am failing, I believe that everybody knows I am failing.”* Research has reported a mixed picture of the factorial structure of this scale (Sagar and Jowett, 2010). Our CFA showed a one factor second-order structure demonstrated superior model fit compared to alternative models (Table 1), echoing the finding in the meta-analysis by Sagar and Jowett (2010). However, item 16 (*When I am failing, I hate the fact that I am not in control of the outcome*) and item 12 (*When I am failing, I am not worried about it affecting my future plans*) were dropped in the subsequent analysis due to factor loadings (item 16 = 0.41; item 12 = 0.40) below the 0.50 cut-off recommended by Kline (2016). The scale has an alpha value of 0.94 in the current sample.

**TABLE 1:** Measurement model comparison.

**Model**	**χ^2^**	***df***	**χ^2^ */df***	**CFI**	**TLI**	**RMSEA (90% CI)**	**SRMR**	**Dχ^2^(D*df*)**
Model A	6921.29	2,079	3.33	0.44	0.43	0.11 (0.11, 0.11)	0.12	–
Model B	3863.73	2,064	1.87	0.79	0.79	0.07 (0.06, 0.07)	0.07	3057.56*** (15)
Model C	3550.15	2,059	1.72	0.83	0.82	0.06 (0.06, 0.06)	0.07	313.58*** (5)
Model D	3458.15	2,034	1.70	0.84	0.83	0.06 (0.06, 0.06)	0.07	92 (25)
Model CMV	2803.29	1,689	1.66	0.87	0.86	0.06 (0.05, 0.06)	0.07	746.86 (374)^a^

***p < 0.001.

*χ^2^, chi-square; df, degrees of freedom; CFI, comparative fit index; TLI, Tucker–Lewis index; RMSEA, root mean square error of approximation; SRMR, standardized root mean squared residual; Dχ^2^(D*df*), changes in chi-square and degrees of freedom.*

*Model A: All indicators loaded on a single factor.*

*Model B: Six factors model with fixed mindset, learning GO, prove GO, avoid GO, impostor phenomenon, and fear of failure (single factor).*

*Model C: 11 factor model with fixed mindset, learning GO, prove GO, avoid GO, impostor phenomenon, fear of failure (second order with five first order subdimensions: fear of experiencing shame and embarrassment, fear of devaluing one’s self-estimate, fear of having an uncertain future, fear of important others losing interest, and fear of upsetting important others.*

*Model D: 10 factor model with fixed mindset, learning GO, prove GO, avoid GO, impostor phenomenon, fear of experiencing shame and embarrassment, fear of devaluing one’s self-estimate, fear of having an uncertain future, fear of important others losing interest, and fear of upsetting important others.*

*Model CMV: 12 factor model, Model C with an additional common method factor.*

*^a^Model C compared to Model CMV.*

#### Goal Orientation

Goal orientation was measured using the 13-item Work Domain Goal Orientation Instrument (Brett and Vandewalle, 1999) which comprises three subscales. Research shows all three dimensions have good internal consistency, exceeding α = 0.75 (Brett and Vandewalle, 1999) and test-retest reliability exceeding α = 0.57 (Vandewalle, 1997). Four items measure participants’ desire to prove and gain favorable judgments for their competence (prove GO), such as “*I like to show that I can perform better than my coworkers*.” Four items measure participants’ desire to avoid showing incompetence and avoid negative judgments about their competence (avoid GO), such as “*I prefer to avoid situations at work where I might perform poorly*.” Five items measure participants’ desire to improve their competence, develop themselves through the attainment of new skills and master new situations (learning GO), such as “*I enjoy challenging and difficult tasks at work where I’ll learn new skills*.” For each item, participants used a 7-point Likert scale ranging from “strongly disagree” to “strongly agree” to indicate their agreement with each statement. The higher the score for each subscale, the more the participant endorses the respective disposition reflected. In line with previous research (Brett and Vandewalle, 1999), our CFA supported a three-dimensional structure with good internal consistency in each of the subscales (α = 0.87 learning GO; α = 0.84 prove GO; and α = 0.90 avoid GO).

#### Impostor Phenomenon

Impostor phenomenon was measured using the 20-item Clance Impostor Phenomenon Scale (CIPS) (Clance, 1985). The CIPS has high internal consistency α = 0.92 (Chrisman et al., 1995). It comprises three subscales: the Fake subscale assesses self-doubt and concerns about intelligence and ability; the Discount subscale assesses thoughts about the inability to acknowledge good performance and praise for performance; the Luck subscale assesses thoughts of having accomplished tasks due to luck, chance, or even error, as opposed to ability (French et al., 2008). Participants were asked to indicate how true each statement is of them using a 5-point Likert scale ranging from “not at all true” to “very true.” Examples statements are: “*At times, I feel my success has been due to some kind of luck”* and “*Sometimes I’m afraid others will discover how much knowledge or ability I really lack*.” In line with the systematic review by Mak et al. (2019) our CFA supports a unidimensional structure of this scale. However, item 1 (*I have often succeeded on a test or task even though I was afraid that I would not do well before I undertook the task*) and item 2 (*I can give the impression that I’m more competent than I really am*), item 19 (*If I’m going to receive a promotion or gain recognition of some kind, I hesitate to tell others until it is an accomplished fact*) and item 20 (*I feel bad and discouraged if I’m not “the best” or at least “very special” in situations that involve achievement*) were dropped in the subsequent analysis due to low factor loadings (item 1 = 0.18; item 2 = 0.28; item 19 = 0.34; item 20 = 0.41). The scale showed good internal consistency (α = 0.91) in the current sample.

### Control Variables

We controlled for gender due to the significant differences reported by Kumar and Jagacinski (2006) concerning mindset, impostor phenomenon and goal orientation in their study. Gender is also of specific interest in relation to impostor feelings, as sixteen of thirty-three studies included in a meta-analysis reported women had higher impostor feelings than men, whilst the remaining seventeen studies showed no significant gender differences (Bravata et al., 2020). Research suggests female faculty and students experience more impostor feelings than male faculty and students, and than females in other domains (Howe-Walsh and Turnbull, 2016; Vaughn et al., 2020; Muradoglu et al., 2021). This may be a function of context as a result of women occupying a minority in the higher education faculty (Howe-Walsh and Turnbull, 2016; Parkman, 2016; Vaughn et al., 2020; Muradoglu et al., 2021). Which emphasizes “brilliance” in the form of raw intellectual talent which in turn exacerbates feelings of intellectual fraudulence in women (Muradoglu et al., 2021). Conversely, female leaders exhibit less impostor feelings (Sanford et al., 2015).

We also controlled for age as a meta-analysis revealed mixed effects for age and impostor feelings. Two studies found impostor feelings diminished with age, three found no effect, and one found age negatively related to impostor feelings in professionals, yet not undergraduates (Bravata et al., 2020). This was theorized as being attributable to the much smaller undergraduate age range. Further, Sanford et al. (2015) reported no relationship between CIPS scores and age in female leaders; although their thematic analysis did reveal a relationship between more impostor feelings for the leaders who felt lacking in age.

Lastly, we controlled for participants tenure in role, as research suggests that impostor phenomenon is experienced more frequently by those new to a position and diminishes with experience (Kets de Vries, 2005; Sanford et al., 2015). Sanford et al. (2015) found impostor feelings were related to inexperience and the women who lacked impostor feelings expressed experience enabled them to gain confidence. Vaughn et al. (2020) and Muradoglu et al. (2021) reported junior academics experienced stronger impostor feelings than senior faculty. However, Vaughn et al. (2020) also reported moderate to intense levels of impostor phenomenon across positions and time in their sample of female academics.

### Statistical Analyses Strategy: Two-Step Structural Equation Modeling

We conducted SEM using the maximum likelihood estimator of STATA (v16) to investigate our proposed serial-parallel mediation latent structure model (Figure 1). SEM is considered an appropriate approach that offers an advantage over conventional regression methods for our study as it enables the simultaneous estimation of complex relationships between latent constructs (Kline, 2016). For ease of identifying specification errors and reducing the potentials of interpretational confounding (Burt, 1976), we followed the two-step SEM modeling procedure proposed by Anderson and Gerbing (1988). In the first step, we performed CFA comparing four alternative models to validate the factorial structure and the distinctiveness of the latent study variables. We then continued to estimate the proposed structural model (Figure 1) in step two using a full SEM function. We consulted a range of goodness of fit indicators including *χ*^2^, degrees of freedom, comparative fit index (CFI), Tucker–Lewis index (TLI), root mean square error of approximation (RMSEA), and standardized root mean squared residual (SRMR) to assess the overall model fit. To decompose this serial-parallel mediation model (Hayes, 2017) we used PROCESS v3.5 macro with 5000 bootstrapped confidence intervals.

## Results

### Measurement Structure and Discriminant Validity Testing

We tested four alternative measurement models to validate the factorial structures and the distinctiveness of the study variables in the current sample. As shown in Table 1, the single-factor model (Model A) demonstrated a poor fit across all goodness of fit indexes reported and its *χ*^2^ value is significantly different from and considerably larger than all three alternative models. This shows good distinctiveness between the key constructs. Due to the mixed picture of the factorial structure of fear of failure reported in the literature (Sagar and Jowett, 2010), three remaining CFA models comprising the same latent factors of fixed mindset, learning GO, prove GO, avoid GO, impostor phenomenon but with alternative factorial structures of fear of failure were compared. The results showed that Model C with a second-order structure of fear of failure demonstrated a better fit across all goodness of fit indexes compared to Model B, which comprised a unidimensional form of fear of failure. Model C also shows a significantly smaller *χ*^2^ value than Model B. Moreover, Model D with a five sub-dimensions structure of fear of failure demonstrated marginal advantage over Model B in terms of the reported goodness of fit indexes and an non-significant reduction of *χ*^2^ value at the cost of 25 degrees of freedom. As the meta-analysis by Sagar and Jowett (2010) also supplied validity evidence for a second-order structure of fear of failure, we have therefore decided to adopt this parsimonious and better-performing Model C in the subsequent analysis. Four items from impostor phenomenon and two items from fear of failure were removed due to low factor loadings ranging between 0.18 and 0.44. All remaining items have factor loadings that fell between 0.53 and 0.97, which is higher than the widely accepted threshold of 0.50 recommended by Kline (2016).

### Common Method Variance Analysis

Due to the self-report nature of data collection, as part of the pre-analysis, we examined the common method variance in the current sample. Harman’s single-factor test using Principal Axis Factoring extraction showed that only 28.8% variance can be explained by a common factor which is lower than 50% threshold (Malhotra et al., 2006). To verify this result, we also conducted a common latent factor analysis suggested by Podsakoff et al. (2003). Based on Model C identified in the previous CFA procedure, we loaded all indicators on a common method latent factor, in addition to their respective theoretical constructs, to build a common method variance (CMV) model. As shown in Table 1, the CMV model exhibited no advantage in terms of goodness of fit over Model C and the common method factor explained only 3% of the variance in the indicators, which accounts for a small effect size (Cohen, 1992). Therefore, common method variance did not appear to pose a serious concern for the current study.

### Descriptive Statistics and Correlation Analysis

As shown in Table 2, fixed mindset is positively correlated with fear of failure (*r* = 0.25, *p* < 0.001), avoid GO (*r* = 0.21, *p* < 0.001) impostor phenomenon (*r* = 0.22, *p* < 0.001), and negatively correlated with learning GO (*r* = −0.22, *p* < 0.001). Fear of failure is positively correlated with prove GO (*r* = 0.24, *p* < 0.001), avoid GO (*r* = 0.50, *p* < 0.001) and impostor phenomenon (*r* = 0.68, *p* < 0.001). Learning GO is positively related to prove GO (*r* = 0.23, *p* < 0.001), negatively related to avoid GO (*r* = −0.40, *p* < 0.001), and impostor phenomenon (*r* = −0.15, *p* < 0.001). Prove GO shares a positive correlation with avoid GO (*r* = 0.22, *p* < 0.001) and impostor phenomenon (*r* = 0.21, *p* < 0.001). Avoid GO is positively correlated with impostor phenomenon (*r* = 0.51, *p* < 0.001).

**TABLE 2:** Descriptive statistics and correlations (*n* = 201).

		**1**	**2**	**3**	**4**	**5**	**6**	**7**	**8**	**9**
1	Age	−								
2	Gender^a^	–0.01	−							
3	Role^b^	0.51***	0.08	−						
4	Fixed mindset	–0.14	0.18*	–0.10	0.95					
5	Fear of failure	−0.26***	0.07	−0.16*	0.25***	0.94				
6	Learning GO	–0.05	0.09	0.04	−0.022**	–0.13	0.87			
7	Prove Go	−0.29***	0.10	–0.12	0.01	0.24**	0.23**	0.84		
8	Avoid GO	−0.19**	0.08	−0.16*	0.21**	0.50***	−0.40***	0.22**	0.90	
9	Impostor phenomenon	−0.38***	–0.02	−0.25***	0.22**	0.69***	−0.15*	0.21**	0.51***	0.94

***Mean***		40.43	−	4.51	3.00	2.88	5.71	4.72	3.25	2.75
***SD***		12.11	−	6.08	1.11	0.81	0.89	1.27	1.42	0.86

*p < 0.05, **p < 0.01, ***p < 0.001 (two tailed). Cronbach’s alpha values are on the diagonal line. ^*a*^Gender is coded as female = 0, male = 1. ^*b*^Role: Number of years worked in the current role.

As for the covariates, males are found to report a higher level of fixed mindset on average than females (*r* = 0.18, *p* < 0.01). The longer (in years) someone worked in the current role, the less they reported fear of failure (*r* = −0.16, *p* < 0.01), avoid GO (*r* = −0.16, *p* < 0.01), and impostor phenomenon (*r* = −0.25, *p* < 0.001). It was also found that age is negatively correlated with fear of failure (*r* = −0.26, *p* < 0.001), prove GO (*r* = −0.29, *p* < 0.001), avoid GO (*r* = −0.20, *p* < 0.001) and impostor phenomenon (*r* = −0.38, *p* < 0.001).

### Hypothesis Testing

**FIGURE 2:**
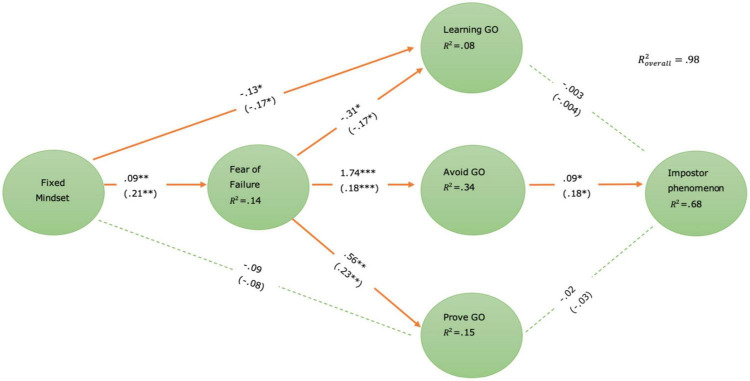
Direct effects of the serial-parallel SEM Model (standardized coefficients in parentheses). Dashed line represents non-significant effects. ^∗^*p* < 0.05, ^∗∗^*p* < 0.01, ^***^p < 0.001 (one tailed).

We proceeded with the second step to investigate the full latent factor structural model. As illustrated in Figure 2, our model showed a good fit with the data [*χ*^2^ = 2473.09, *df* = 1, 771, *χ*^2^/*df* = 1.40, RMSEA = 0.05, 90% CI (0.04, 0.05), CFI = 0.92, TLI = 0.91, SRMR = 0.07].

#### Simple Relationships

The results in Table 3 show the simple relationships between the latent variables without considering any of the indirect effects. Fixed mindset had a significant and positive effect on impostor phenomenon (β = 0.16, *p* = 0.024), supporting **H1**. The total effect of fixed mindset on fear of failure was also found to be significant and positive (β = 0.21, *p* = 0.008), supporting **H2.** As for the three types of goal orientations, the results showed that fixed mindset was significantly and negatively associated with learning GO (β = −0.21, *p* = 0.007) and positively and significantly associated with avoid GO (β = 0.17, *p* = 0.025), supporting **H3a** and **H3c**. There was a very small non-significant negative relationship between fixed mindset and prove GO (β = −0.03, *p* = 0.673). **H3b** is therefore not supported. Fear of failure was found to be significantly and positively related to impostor phenomenon (β = 0.75, *p* < 0.001), supporting **H4**. Among the three types of goal orientations, only avoid GO was found to have a positive and significant relationship with impostor phenomenon (β = 0.18, *p* = 0.019), supporting **H5c**. Learning GO (β = 0.00, *p* = 0.95) and prove GO (β = −0.03, *p* = 0.626) were not found to have significant relationships with impostor phenomenon, **H5a** and **H5b** were thus not supported. Fear of failure was found to have a significant and negative relationship with learning GO (β = −0.17, *p* = 0.042), supporting **H6a**. A positive association between fear of failure and prove GO was found (β = 0.23, *p* = 0.009), supporting **H6b**. A positive and significant relationship between fear of failure and avoid GO was also found in the current sample (β = 0.56, *p* < 0.001), supporting **H6c**.

**TABLE 3:** Unstandardized coefficients of the simple relationships between variables.

**Hypothesis**	**Path**	***b***	***SE***	***p***
H1	Mindset → Impostor phenomenon	0.11	0.05	0.024
H2	Mindset → Fear of failure	0.09	0.03	0.008
H3a	Mindset → Learning GO	–0.16	0.06	0.007
H3b	Mindset → Prove GO	–0.03	0.08	0.673
H3c	Mindset → Avoid GO	0.23	0.10	0.025
H4	Fear of failure → Impostor phenomenon	1.15	0.07	<0.001
H5a	Learning GO → Impostor phenomenon	0.00	0.05	0.95
H5b	Prove GO→ Impostor phenomenon	–0.02	0.04	0.626
H5c	Avoid GO → Impostor phenomenon	0.09	0.04	0.019
H6a	Fear of failure → Learning GO	–0.31	0.15	0.042
H6b	Fear of failure → Prove GO	0.56	0.21	0.009
H6c	Fear of failure → Avoid GO	1.74	0.34	<0.001

#### Indirect Effects

The SEM showed that there was a significant and positive indirect effect between fixed mindset and impostor phenomenon (β = 0.17, *p* = 0.007). We further deconstructed this serial-parallel mediation mechanism into seven indirect paths using PROCESS v3.5 with 5000 bootstrapping method (Hayes, 2018). The results in Table 4 show that the relationship between fixed mindset and impostor phenomenon was significantly mediated by fear of failure [β = 0.11, (0.05, 0.18)], supporting **H7a**. None of the three types of goal orientations, learning GO [β = −0.0003, (−0.03, 0.03)], prove GO [β = 0.001, (−0.01, 0.01)] and avoid GO [β = 0.02, (−0.01, 0.06)], were found to be a significant mediator between fixed mindset and impostor phenomenon. Hypotheses **H7b**, **H7c** and **H7d** were thus not supported. The serial mediation paths through fear of failure and learning GO [β = 0.00, (−0.004, 0.004)], and fear of failure and prove GO [β = −0.0006, (−0.005, 0.005)] were not found to be significant. **H8a** and **H8b** were not supported. The only significant serial mediation path in the relationship between fixed mindset and impostor phenomenon was through fear of failure and avoid GO [β = 0.02, (0.004, 0.04)], supporting **H8c**. The indirect effect of fear of failure was found to be significantly stronger than the serial mediation path through fear of failure and avoid GO [β = 0.09, (0.03, 0.16)], demonstrating that fear of failure had the strongest explanatory role in the relationship between fixed mindset and impostor phenomenon. It is worth noting, although avoid GO was not found to be a significant mediator in the current sample, the indirect effect of avoid GO was not considerably weaker compared to the serial path through fear of failure and avoid GO [β = −0.003, (−0.04, 0.03)]. Considering this negligible size difference and the problems associated with a binary view of statistical siginificance, it is not prudent to completely disregard the potential explanatory role of avoid GO on the indirect relationship between fixed mindset and impostor phenomenon. Indeed, due to the limitation of the current sample size, this study was underpowered for the testing of small effects based on Kline’s (2016) rule of thumb.

**TABLE 4:** Decomposing the indirect effect of Mindset on Impostor Phenomena in the serial-parallel mediation model (5000 bootstrapped).

**Hypothesis**	**Indirect path**	**Unstandardized coefficient**	**95% CI Lower**	**95% CI Upper**
	Total indirect effect	0.12	0.05	0.19
H7a	Mindset → Fear of failure → Impostor phenomenon	0.09	0.04	0.15
H7b	Mindset → Learning GO → Impostor phenomenon	−0.0002	–0.02	0.02
H7c	Mindset → Prove GO → Impostor phenomenon	0.001	–0.01	0.01
H7d	Mindset → Avoid GO → Impostor phenomenon	0.01	–0.01	0.05
H8a	Mindset → Fear of failure → Learning GO → Impostor phenomenon	0	–0.003	0.003
H8b	Mindset → Fear of failure → Prove GO → Impostor phenomenon	−0.0005	–0.004	0.004
H8c	Mindset → Fear of failure → Avoid GO → Impostor phenomenon	0.02	0.003	0.03
	Indirect path H7a minus indirect path H8c	0.07	0.03	0.13
	Indirect path H7d minus indirect path H8c	−0.002	–0.03	0.03

## Discussion

This study had two aims. First, to investigate the relationships between mindset, fear of failure, the three types of goal orientations and impostor phenomenon. Second, to investigate the potential mediating roles of fear of failure and three types of goal orientations in the indirect mechanism between mindset and impostor phenomenon. We are the first, to our knowledge, to connect and explore the nature of these relationships by deconstructing them using a serial-parallel mediation model, in the work domain.

### Fixed Mindset and Impostor Phenomenon

We found a small effect of mindset on the impostor phenomenon, supporting **H1**. The results suggest that people who do not believe that they can improve their abilities, tend to feel more like an impostor in the work domain. These findings support the relationship between fixed mindsets and impostor feelings reported by Kumar and Jagacinski (2006). However, in contrast to Kumar and Jagacinski (2006), who only found a link between a fixed mindset and impostor feelings within female undergraduates, there were no gender differences in our sample of professionals. Our findings provide support for the proposition that fixed mindsets and impostors are related due to a shared fixed view of ability as proposed by Kumar and Jagacinski (2006).

### Mindset and Fear of Failure

A slightly larger but still small positive effect of mindset on fear of failure was also found, suggesting that the less someone believes they can improve their abilities and grow, the more frequently they will experience fear of failure in the workplace, supporting **H2**. This finding aligns with research demonstrating the influential role that one’s beliefs about their abilities (mindset) plays in their relationship with failure; and with research highlighting that mindset predicts subsequent efforts following failures (Hong et al., 1999; Blackwell et al., 2007).

### Mindset and Goal Orientation

The results showed that mindset had a small negative effect on learning GO, supporting **H3a**. This suggests that a lack of belief in one’s ability to develop and grow (fixed mindset) is associated with a lower level of learning GO. Thus, the reverse is also true. A higher belief in one’s ability to develop and grow (growth mindset) is associated with a higher level of learning GO. This finding provides empirical evidence for a direct relationship between mindset and learning GO and supports prior research highlighting this (Dweck and Leggett, 1988; Cury et al., 2006).

**H3b** was not supported in this study, contradicting earlier research (Dweck and Leggett, 1988; Cury et al., 2006), and supporting the questions raised by Vandewalle et al. (2019) regarding the lack of consistent empirical evidence linking mindset to the performance goal orientations. Intrigued, we took a closer look at the results which revealed a small but positive and significant indirect relationship between fixed mindset and prove GO via fear of failure in our structural model. We found that fear of failure served as a suppressor which completely mediated this indirect path, reversing it in the positive direction [β = 0.06, BCa 95% CI (0.02, 0.12)]. In other words, the relationship between mindset and prove GO was hiding behind the suppression effect (Conger, 1974; Cohen et al., 2013) of fear of failure. Meaning, when someone has a fixed view of their abilities, and fear of failure is also present, they are more likely to be motivated by a desire to prove their abilities to others in the workplace. This finding may be explained by the prove GO being motivated by both fear of failure and need for achievement (Elliot and Church, 1997). These dual motivating drives may result in different choices and outcomes, depending on context and which of these drivers are most salient at the time. Although this was not one of our original hypotheses, we believe it is an important finding as it partially supports **H3b,** by revealing the indirect nature of the association between fixed mindset and prove GO. Further, it emphasizes the pivotal motivating effect of fear of failure in this mechanism. Moreover, the suppression effect of fear of failure observed in our sample contributes to demystifying the inconsistent evidence regarding the relationship between mindset and goal orientation highlighted by Vandewalle et al. (2019).

We also observed a significant and positive effect of fixed mindset on avoid GO, supporting **H3c**. This effect was small but of comparable size to the effect of fixed mindset on learning GO. This finding aligns with prior research demonstrating performance goal orientations are related to having a fixed view of one’s abilities (Dweck and Leggett, 1988; Cury et al., 2006).

### Fear of Failure and Impostor Phenomenon

Fear of failure was found to be the single most important predictor of impostor phenomenon, compared to all the other predictors tested in this study, supporting **H4**. This strong negative effect demonstrates that the more someone is afraid of failing to perform well at work, the more likely they will feel like an impostor. This finding is consistent with the core theoretical argument that fear of failure is a key motivator for impostors (Clance and O’Toole, 1987) and aligns with research connecting the two constructs (Cozzarelli and Major, 1990; Thompson et al., 1998; Howe-Walsh and Turnbull, 2016; Neureiter and Traut-Mattausch, 2017). Further, it provides support to research demonstrating that impostor thoughts relate to social anxiety via fear of negative evaluation, along with an impostor’s needs for social recognition and over-concern with what others think of them (Chrisman et al., 1995; Thompson et al., 2000).

### Goal Orientation and Impostor Phenomenon

Among the three types of goal orientations, only the avoid GO was significantly correlated with impostor phenomenon, **H5a** and **H5b** were thus not supported. The non-significant relationship found between learning GO and impostor phenomenon in this study runs counter to the significant negative relationship found in females by Kumar and Jagacinski (2006). Further, the non-significant effect of prove GO on impostor phenomenon also contradicts the significant relationship found by Kumar and Jagacinski (2006) between the desire to want to prove one’s abilities to others and impostor fears. We speculate this could be due to the differences between the samples. In Kumar and Jagacinski’s (2006) study undergraduates with a median age of 19 were utilized, whereas our study utilized a range of working professionals with a median age of 40. Kumar and Jagacinski (2006) reported gender differences for goal orientation and impostor feelings in their study, whereas we found no gender differences in our study.

Avoid GO on the other hand was found to have a small but significant positive effect on impostor phenomenon, supporting **H5c**. The finding showed that the more someone is motivated by the desire to avoid disproving competence to others and avoid negative judgments about their competence (Vandewalle, 1997), the more they will experience impostor thoughts and feelings. Thus, these findings highlight that the desire to avoid shows of inability and the desire to protect oneself from judgments about their abilities, are significant motivators for impostors. This supports Kumar and Jagacinski (2006) findings that linked avoid GO with impostor fears in male undergraduates and expands on this by highlighting its prevalence in both professional men and women. Further, these findings support research proposing impostor fears are rooted in fears of social exposure and attracting negative judgments from others (Thompson et al., 2000).

### Fear of Failure and Goal Orientation

Fear of failure had small to strong associations with all three types of goal orientation. Specifically, we found a small but significant negative correlation between fear of failure and learning GO, supporting **H6a**. Frequent experience of fear of failure in the workplace is associated with a decreased likelihood of being motivated by the desire to improve one’s abilities, acquire new skills or achieve mastery of new situations. Thus, the reverse can also be stated. Our results suggest, the more someone is motivated by a learning GO in the workplace, the less fear of failure they are likely to experience. This aligns with research demonstrating that the learning GO is not motivated by fear of failure and supports the suggestion that a learning GO is qualitatively different to the performance goal orientations (Elliot and McGregor, 1999). However, it contradicts the null relationship found by prior research between learning GO and fear of failure (Elliot and McGregor, 1999).

A slightly stronger but positive relationship between fear of failure and prove GO was found, supporting **H6b.** This suggests that as fear of failure increases in the workplace, the more likely someone will be motivated to prove their abilities and demonstrate their competence to others. This finding aligns with research demonstrating that fear of failure is one of the core drivers for the prove GO (Elliot and Church, 1997); and that the two are positively related (Conroy and Elliot, 2004).

The association between fear of failure and avoid GO was found to be the second strongest of all the relationships explored in this study, supporting **H6c**, suggesting that increased fear of failure in the workplace is associated with an increased desire to avoid showing one’s incompetence to others. This finding supports literature reporting that fear of failure is an antecedent of avoid GO (Conroy and Elliot, 2004).

### Covariates

The one interesting finding for gender was that males were more likely to have a fixed view of their abilities (fixed mindset) than females. Our findings contradict Kumar and Jagacinski (2006) who reported women were more likely to have a fixed view of their abilities, alongside more impostor feelings and different goal orientations than men. These differences may be attributed to limitations in our sample size, or differences between the sample populations, i.e., professionals versus undergraduates, and significant age differences. In reference to the last point, Kumar and Jagacinski reported a median age of 19 years, to allow for comparison, the median (and mean) age in our study was 40 years.

Our research suggests professional women do not experience greater impostor feelings than men, thus supporting research reporting similar findings (Sanford et al., 2015; Bravata et al., 2020), and lending weight to the proposal that increased impostor feelings in women are a function of the contexts which exacerbate such feelings. This becomes evident when women are the minority and when intellectual talent is prized, as is the case for female academics (Howe-Walsh and Turnbull, 2016; Parkman, 2016; Vaughn et al., 2020; Muradoglu et al., 2021).

Negative relationships were highlighted in the correlation analysis between time in role and age, with impostor feelings, fear of failure and the performance goal orientations. This supports research that has found, that as age, time in role and experience increases, impostor fears diminish (Kets de Vries, 2005; Sanford et al., 2015; Bravata et al., 2020). However, as highlighted by Vaughn et al. (2020) moderate to intense levels of impostor feelings were experienced across position and time in their sample of female academics. Therefore, although diminishing, it remains important not to discount the impact of impostor fears on more experienced populations.

Interpreted collectively, our findings suggest there is a natural reduction that happens over time, in feelings of fraudulence, fears of failing and drives to avoid showing inability or prove oneself to others. We hypothesize that this is brought about through learning and experience which provides the development of skills and competence; which can, in turn, lead to increases in self-identity and self-efficacy. Our results suggest that as people develop and grow over the years, their experience can reduce their fear of being “found out,” their fear of failure; and their desire to prove themselves or avoid showing inability to others.

### Indirect Effects

We deconstructed the indirect effect between fixed mindset and impostor phenomenon into four sets of simple mediations and three sets of serial mediations. The results showed two significant indirect paths: the simple mediation path through fear of failure, and the serial mediation path through fear of failure and avoid GO. Specifically, we observed that as fixed mindset increases one unit, impostor phenomenon will increase 0.11 units via the indirect effect of fear of failure. Although the effect is small, fear of failure explained 75.33% of the total indirect effect of fixed mindset on impostor phenomenon, supporting **H7a**. This indirect path was connected by the relatively strong association observed between fixed mindset, fear of failure and the impostor phenomenon. This first significant indirect pathway is in line with previous research linking fixed mindsets to impostor fears (Kumar and Jagacinski, 2006); mindset and fear of failure (Hong et al., 1999; Blackwell et al., 2007); fear of failure and impostor feelings (Cozzarelli and Major, 1990; Thompson et al., 1998).

None of the three types of goal orientation were significant mediators on the indirect path between fixed mindset and impostor phenomenon, thus **H7b, H7c, and H7d** were not supported. The indirect paths through learning GO and prove GO only accounted for 0.2 and 0.73% of the total indirect effect, respectively. This was unsurprising, as we found no significant effect of learning GO and prove GO on the impostor phenomenon.

Contrary to our expectation, given the significant associations observed between avoid GO and both fixed mindset and impostor phenomenon, avoid GO was not found to be a significant mediator. It is worth noting that although non-significant, this simple indirect path still accounted for 11.37% of the total indirect effect – a relatively comparable size as the significant serial mediation path via fear of failure and avoid GO (shown in Table 4). We caution against completely disregarding the potential explanatory role of avoid GO on the association between fixed mindset and impostor phenomenon based on a risky binary view of statistical significance and a limited sample size. It is therefore suggested that future research should replicate the current study to investigate the mediating role of avoid GO using a larger sample size.

In terms of the three serial mediation paths examined, the coefficient of the serial indirect path through fear of failure and learning GO was found to be zero and the indirect path through fear of failure and prove GO accounted for a mere 0.4% of the total indirect effect between fixed mindset and impostor phenomenon. **H8a** and **H8b** were thus not supported. These findings were not unexpected as learning and prove GOs were not related to impostor phenomenon.

The serial mediation path through fear of failure and avoid GO was significant, supporting **H8c**. This serial indirect path was the result of the observed associations between fixed mindset, fear of failure, avoid GO and impostor phenomenon. The second significant indirect serial mediation pathway weaves together research highlighting a connection between fixed mindsets and impostor fears (Kumar and Jagacinski, 2006); with the relationship identified between mindset and fear of failure (Hong et al., 1999; Blackwell et al., 2007); with the finding that avoid GOs are motivated by a fear of failure (Elliot and Church, 1997; Conroy and Elliot, 2004); to Kumar and Jagacinski’s (2006) finding that avoid GO is related to impostor feelings.

Taken together, the indirect path analysis has demonstrated the paramount importance of fear of failure in explaining the relationship between fixed mindset and impostor phenomenon. Specifically, although both indirect paths were significant, the simple mediation path through fear of failure alone was found to be significantly stronger (accounting for 62.1% more variance of the total indirect path) than the serial mediating path through fear of failure and avoid GO.

We conclude, the more individuals believe their abilities are fixed, the more afraid they are of failing and the more like an impostor they are likely to feel. Further, when someone believes their abilities are fixed, fear of failure is salient and they are motivated to avoid showing inability, they are more likely to experience impostor feelings. Conversely, the more people believe their abilities are capable of growth, the less afraid they are of failing, the less likely they will have impostor feelings and the more motivated they will be to improve their abilities. Therefore, cultivating growth mindsets and learning GO, reconceptualizing failure as a normal part of the journey toward success, and making it psychologically safe to fail, could be key levers to reducing the negative outcomes associated with fixed mindsets and impostor feelings.

### Limitations and Future Research

Due to the nature of this study design, we cannot conclude causation, therefore a follow-up longitudinal study would be beneficial to understand causality. Self-report survey data was utilized and thus subject to inherent biases. Future studies could therefore utilize other methods of data collection including objective measures of performance to explore this potentially related outcome. The sample size was relatively small (*N* = 201) although efforts were made to obtain a diverse work sample from different industries. Future research would benefit from increasing the sample size to increase power, which could potentially result in a higher probability of detecting smaller effect sizes that in this study were non-significant, such as the explanatory effect of avoid GO as a sole mediator. Also, considering the ordinal categorical nature of the data, diagonally weighted least squares might be a more suitable estimator for future studies with a larger sample size (Li, 2016).

Finally, longitudinal studies which investigate the impacts of mindset, fear of failure, goal orientation, and impostor feelings, on outcomes such as job performance and wellbeing, would be natural progressions for exploring the interrelationships observed in this study. Further, a possible moderator to explore at the individual and organizational level would be psychological safety, due to the relationship found between unsafe organizations and avoiding failure (Edmondson, 2018). Furthermore, exploring individual differences such as perceived ability, effort attributions, self-esteem, self-efficacy, identity development and neuroticism as moderators could provide greater insight into how the relationships are operating between mindset and impostor phenomenon, via fear of failure and goal orientation. We were unable to analyze organizational or industry effects due to the limited number of participants within the same sector or organization. Future research would benefit from exploring the potential effect of contextual level variables and any possible interaction effects between such variables and gender.

### Implications

Our findings demonstrate that how people see their abilities, and whether they believe they can develop them (mindset), affects their relationship with failure and whether they believe failure defines them (fixed mindset) or provides them with opportunities for growth and development (growth mindset). Their relationship with failure relates to the dispositional goal orientations individuals are motivated to navigate workplace performance and achievement situations with. Namely, the higher the fear of failure, the more likely they will try to prove their ability (prove GO) or avoid showing their inability (avoid GO) to others. The less fear of failure they have, the more likely they are to be motivated to improve their ability (learning GO). Furthermore, increased fear of failure and avoid GOs are related to increased impostor feelings. Thus paradoxically, it appears, if an individual believes their abilities are fixed, is afraid of failing, and wishes to avoid showing inability; the more likely their fear of failure will be fueled, and the more like an impostor they are likely to feel. Our results also illuminate that the learning GO is positively related to growth mindsets, and negatively related to fear of failure and impostor feelings.

Our findings highlight how influential fear of failure is in the workplace. Fear of failure is fueled by a belief that abilities are fixed. It drives motivations to either prove ability or avoid showing inability to others; and ultimately leads to impostor feelings. Our findings also illuminate the potential beneficial mechanisms or “levers” which organizations can use to alleviate fear of failure (and its subsequent outcomes), by disrupting the connections between fixed mindsets and impostor feelings, operating via fear of failure and goal orientation. Namely, as both growth mindsets and learning GO are negatively related to fear of failure, our results suggest it may be possible for organizations to disrupt the key emotional engine of fear of failure, by cultivating growth mindsets and encouraging learning GO in the workplace. Our research suggests this will aid in reconceptualizing failures and make it safer to fail. Thus, breaking the links between fixed mindsets, fear of failure, avoid GO, and impostor feelings.

Our results also suggest that if someone has a predominantly fixed mindset, is experiencing a higher fear of failure, and is motivated to prove their abilities, then this will not lead to impostor feelings. Further, if an individual has a predominantly growth mindset, with lower levels of fear of failure and is motivated to develop their abilities, our results suggest this too will not lead to impostor feelings. Conversely, our findings demonstrate that when fear of failure is present and met with the motivation to avoid shows of inability, this reinforces fear of failure and leads to more impostor feelings. Therefore, whether motivated by wanting to prove oneself (from a fixed mindset) or to develop and grow one’s abilities (from a growth mindset), action rather than avoidance, appears to be key in reducing impostor feelings.

It is important to acknowledge that impostor are often high achieving individuals with no performance deficits, whose fears can motivate them to perform better; thus, supporting them in achieving job mastery and enhancing interpersonal performance at work (Tewfik, 2021). Nevertheless, it is the cost to the impostor’s wellbeing which organizations need to account for. An impostor is more likely to suffer from low self-esteem and other psychological issues, such as depression and anxiety, in addition to a myriad of other challenges in the workplace, such as burnout (Sakulku and Alexander, 2011; Bravata et al., 2020). We believe the findings of this study support the request by Bravata et al. (2020), for evidence-based interventions which can alleviate the psychological impacts of impostor fears and better support those struggling with impostor feelings. Our findings suggest the unique value of cultivating growth mindsets and learning GOs, alongside reconceptualizing failure, to protect individuals from impostor feelings; by reducing their fear of failing and empowering them to grow through and beyond challenges.

### Practical Applications

Our findings suggest that there are three levers that organizations can use to reduce fear of failure and “break” the chain between fixed mindsets and impostor feelings, via fear of failure and the avoid GO.

#### Cultivating Growth Mindsets

The nature of the mediated relationships was predicated by mindset. Thus, our results highlight cultivating growth mindsets could alter the nature of these mediated relationships, by reducing fear of failure. This, in turn, has the potential to reduce the likelihood of impostor feelings, and the motivation to use performance goal orientations. Therefore, the first practical application of this study is cultivating growth mindsets via training and cultural interventions.

The proposed method for cultivating growth mindsets is using a suite of training materials, such as those discussed by Dweck (2017). The purpose of these materials is to illuminate neuroplasticity and the brain’s ability to adapt; therefore, facilitating the capacity for lifelong learning and continuous improvement. These materials helpfully contain myth-busting messages around intelligence being fixed and case studies of those who have operated with growth mindsets and achieved inspiring feats. The culture of the organization also needs to be continually assessed for fixed mindset paradigms and replaced with growth-orientated ones. This will be a particularly important practice for leaders, in terms of the behaviors they role model, the language they use, the expectations they communicate to their followers and how effort, versus ability, is characterized and subsequently attributed. As research shows this is significant for mindset, goal orientation and impostor feelings (Blackwell et al., 2007; Vandewalle et al., 2019; Vaughn et al., 2020). It is of paramount importance to ensure any attempts to embed a growth mindset consider the moderating role of the social environment on the efficacy of the growth mindset intervention. As the effects of interventions are more sustained when peer norms align with messages of the intervention and support taking on challenges (Yeager et al., 2019).

#### Activating/Strengthening a Learning Goal Orientation

Our results showed that growth mindsets are positively related to learning GO. Therefore, the second practical application of this study is activating/strengthening a learning GO. By so doing, it may be possible to reverse-engineer, or rather evoke, a growth mindset. Due to the cross-sectional design of the study, it is not possible to establish the direction/causation of these relationships. It could be that the relationship between mindset and goal orientation is bi-directional, which would make it possible to create upward spirals of growth, through an interplay of cultivating growth mindsets and consciously activating/strengthening learning GOs. Further, it is proposed, that activating a learning GO could be utilized as a “protective buffer.” To prevent impostor feelings.

A strong learning goal orientation is beneficial for several distal outcomes important to the work domain, including performance, leadership development and style, wellbeing, openness, adjustment to change, and organizational citizenship behaviors (Vandewalle et al., 2019). Importantly, it is possible to train people to utilize learning goal orientations and learning goals (Stevens and Gist, 1997; Kozlowski et al., 2001; Van Hooft and Noordzij, 2009; Noordzij et al., 2013). Further, Vandewalle et al. (2019) have outlined effective strategies, based on prior research, that leaders can use to promote and strengthen a learning goal orientation amongst their employees.

Research has demonstrated the importance of effective goal setting. Namely, high learning goals can mask performance differences resulting from goal orientation, and additionally, enhance a dispositional learning GO (Seijts et al., 2004). Therefore, effective goal setting which focuses on the use of high learning goals may be a potential route to cultivating learning GO, and consequently a growth mindset. An important caveat is that goal setting researchers suggest the use of high learning goals is only more beneficial when ability/skill development is required and suggest when no learning and only motivation is required, a performance goal would be more beneficial (Seijts et al., 2004). Seijts et al. (2004) suggest learning goals are of most benefit to the work domain for roles and environments prone to rapid changes, thereby requiring continual learning of new effective strategies. It could be said, at the time of writing this, when COVID-19 has caused unprecedented disruptions and changes to organizations and the everyday working life of millions of people across the globe, that learning goals and learning GOs have never been more needed.

#### Reconceptualizing Failure – The Safety to Fail

A third crucial practical application of this study is the paramount importance of leaders and organizations reconceptualizing failure and cultivating the safety to fail. The emotional reaction of fear of failure was the driving force behind the relationships between fixed mindsets, impostor feelings and performance goal orientations in this study. The results from the Yeager et al. (2019) study which explored the efficacy of growth mindset interventions, can be viewed in light of the pivotal role that fear of failure plays. “Supports taking on challenges” may relate to an environment where it is safe to fail; thus reducing fear of failure. If true, when social environments support taking on challenges, this may reduce fear of failure and promote greater efficacy of interventions. The critical role of support for taking on challenges may also be alluding to another important moderator – psychological safety. Edmondson (2018) outlines that if a leader does not make it explicitly safe to fail, their people will seek to avoid failure; and avoidance of failure is a characteristic of an unsafe organization.

Our findings suggest that changing one’s relationship with failure, by reconceptualizing it and making it safer to fail, is key to disrupting the relationship between fixed mindsets and impostor feelings, operating via fear of failure and avoid GOs. There is an enormous comfort to be felt when one realizes that those who have succeeded the most, have indeed, failed the greatest number of times. It is how one characterizes and responds to setbacks; and how one attributes and employs effort to grow *through* failures, which ultimately result in success. It is not in spite of, but because of failures, that abilities are developed. When this is truly understood, failure can be viewed as something to be sought after, learnt from, and used to propel one forward, not something to shield against or shy away from. When there is a willingness to learn and grow, through and beyond failures, they become a necessary component along the pathway to success, not the opposite of it (Goalcast, 2018). Viewed through this frame, failures are not the end, they are the beginning.

## Conclusion

These findings suggest mindset is connected to impostor phenomenon via fear of failure and a performance avoid GO in the work domain. Mindset fuels the nature of these connections through its relationship with fear of failure, and fear of failure is the true driving force connecting these relationships. Based on our findings it can be said that cultivating growth mindsets, activating/strengthening a learning GO, and reconceptualizing failure to create the safety to fail, could reduce fear of failure and disrupt these chains of connection. However, further research is necessary to fully understand the picture which connecting these dots has made.

## Data Availability Statement

The raw data supporting the conclusions of this article will be made available by the authors, without undue reservation.

## Ethics Statement

This study involving human participants was reviewed and approved by the Division of Psychiatry and Applied Psychology Ethics Subcommittee of the Faculty of Medicine and Health Sciences at the University of Nottingham. The participants provided their written informed consent to participate in this study.

## Author Contributions

RN produced the initial manuscript as part of her MSc in Occupational Psychology and was responsible for design, data collection, and data interpretation and write-up. AS was Rebecca’s project supervisor and was instrumental at every stage of the manuscript, including, design, data collection, and data interpretation and write-up. WW performed the data analysis, interpreted the results and wrote about them in the final manuscript. All authors contributed to the article and approved the submitted version.

## Conflict of Interest

The authors declare that the research was conducted in the absence of any commercial or financial relationships that could be construed as a potential conflict of interest.

## Publisher’s Note

All claims expressed in this article are solely those of the authors and do not necessarily represent those of their affiliated organizations, or those of the publisher, the editors and the reviewers. Any product that may be evaluated in this article, or claim that may be made by its manufacturer, is not guaranteed or endorsed by the publisher.

## Footnotes

1 Simple relationships is a term adopted from Field (2018) to refer to the simple relationship between two variables without considering any possible indirect effect between these two variables.
